# Hospitalização por Infarto Agudo do Miocárdio: Um Registro de Base Populacional

**DOI:** 10.36660/abc.20190573

**Published:** 2020-11-01

**Authors:** Leonardo Alves, Carisi Anne Polanczyk

**Affiliations:** 1 Universidade Federal do Rio Grande do Sul Porto AlegreRS Brasil Universidade Federal do Rio Grande do Sul, Porto Alegre, RS – Brasil; 2 Universidade Federal do Rio Grande Rio GrandeRS Brasil Universidade Federal do Rio Grande, Rio Grande, RS - Brasil; 3 Hospital de Cardiologia Hospital Santa Casa do Rio Grande Rio GrandeRS Brasil Hospital Santa Casa do Rio Grande - Hospital de Cardiologia, Rio Grande, RS - Brasil; 4 Hospital de Clínicas de Porto Alegre Porto AlegreRS Brasil Hospital de Clínicas de Porto Alegre, Porto Alegre, RS – Brasil

**Keywords:** Infarto do Miocárdio/mortalidade, Hospitalização, Epidemiologia, Fatores de Risco, Prevenção e Controle, I ntervenção Coronária Percutânea

## Abstract

**Fundamento:**

O infarto agudo do miocárdio com supradesnivelamento do segmento ST (STEMI) é uma das principais apresentações clínicas da cardiopatia isquêmica. Dados de base populacional são relevantes para entendimento contemporâneo da epidemiologia da doença.

**Objetivo:**

Descrever incidência, manejo terapêutico, desfechos clínicos hospitalares e eventos cardiovasculares do primeiro ano de seguimento dos indivíduos hospitalizados por STEMI.

**Métodos:**

Estudo de coorte prospectiva de base populacional com registro consecutivo das hospitalizações por STEMI em uma cidade do Sul do Brasil entre 2011 e 2014. Foram incluídos indivíduos com STEMI que apresentaram sintomas de isquemia miocárdica aguda nas últimas 72 horas. Os valores de p < 0,05 foram considerados significativos.

**Resultados:**

A incidência anual de hospitalizações por STEMI foi de 108 casos por 100.000 habitantes. A incidência ajustada foi maior entre os mais velhos (risco relativo 64,9; IC95% 26,9 – 156,9; *p* para tendência linear < 0,001) e entre os homens (risco relativo 2,8; IC95% 2,3 – 3,3; *p* < 0,001). Ocorreram 530 hospitalizações durante o período avaliado e a taxa de reperfusão foi de 80,9%. A mortalidade hospitalar e a taxa de eventos cardiovasculares em 1 ano foram, respectivamente, 8,9% e 6,1%. Os mais velhos apresentaram maior mortalidade hospitalar (risco relativo 3,72; IC95% 1,57 – 8,82; *p* para tendência linear = 0,002) e mais eventos cardiovasculares em 1 ano (hazard ratio 2,35; IC95% 1,12 – 4,95; *p* = 0,03).

**Conclusão:**

Este registro demonstra abordagem terapêutica e mortalidade hospitalar semelhante às observadas em países desenvolvidos. Entretanto, a taxa de hospitalizações foi maior comparada com esses países.

## Introdução

As doenças cardiovasculares (DCV) são a principal causa de mortalidade em adultos de ambos os sexos em todo o mundo, assim como a causa dominante das mortes prematuras; destas, cerca de 75% ocorrem em países de baixa e média renda.^1^ No Brasil, as DCV, mesmo com tendência para o declínio, também são a principal causa de óbitos em adultos.^2^

A cardiopatia isquêmica é responsável pela maioria das mortes devido às DCV. A Organização Mundial da Saúde (OMS) estima que 17,7 milhões de pessoas morreram por DCV em 2015; destas, 7,4 milhões foram devido à cardiopatia isquêmica que, no Brasil, também é a causa dominante entre as DCV.^2^

O infarto agudo do miocárdio com supradesnivelamento do segmento ST (STEMI) é uma das principais apresentações clínicas da cardiopatia isquêmica, e seu reconhecimento clínico é de fundamental importância para imediata estratégia terapêutica. Estudos mostram que, apesar da diminuição da incidência, a mortalidade por STEMI permanece sem relevante variação.^3^^,^^4^

No Brasil, não há dados de base populacional sobre a taxa de hospitalização por STEMI. Por sua vez, as informações sobre hospitalizações por STEMI, tais como mortalidade e taxa de reperfusão, são provenientes, em sua maioria, de registros que apresentam limitações. Esses registros ficam restritos a um hospital ou, se multicêntricos, não são representativos da população, pois decorrem da amostragem por conveniência (seleção por convite ou voluntária), o que pode enviesar as estimativas. Outras limitações são o recrutamento não consecutivo dos pacientes e critérios restritos de elegibilidade, como a seleção de pacientes com sintomas com duração de até 12 horas, sabendo-se que a apresentação após esse período está associada à maior mortalidade.

Nesse contexto, o objetivo deste estudo é descrever a incidência, a abordagem terapêutica, os desfechos clínicos hospitalares e os eventos cardiovasculares do primeiro ano de seguimento de indivíduos hospitalizados por STEMI em uma região circunscrita do Sul do Brasil. Avaliar essas informações é relevante pela elevada incidência dessa doença e escassez de estudos de base populacional^5^ no Brasil. Além disso, registros são uma maneira efetiva de abordar a implementação das diretrizes clínicas e fontes de dados para gestores, profissionais e pesquisadores da área da saúde.^6^^,^^7^

## Métodos

### Desenho do estudo

Foi conduzido um estudo de coorte prospectiva com registro consecutivo das hospitalizações por STEMI na cidade do Rio Grande (RS) entre janeiro de 2011 e dezembro de 2014. Essa cidade está localizada no Sul do Brasil e compreende cerca de 200 mil habitantes, cuja maioria reside na zona urbana (Censo Demográfico 2010). No Rio Grande, há uma emergência aberta – Hospital de Cardiologia/Santa Casa do Rio Grande – que é o centro de referência no município para tratamento da síndrome coronariana aguda e, consequentemente, para onde todos indivíduos com sintomas sugestivos dessa condição se dirigem ou são encaminhados, tornando o nível de perda de encaminhamento muito baixo. Na cidade, não há linha de cuidado do infarto agudo do miocárdio, de modo que o paciente procura espontaneamente os estabelecimentos de saúde.

### Critérios de elegibilidade

Para serem incluídos no estudo, os indivíduos deveriam preencher os seguintes critérios à admissão hospitalar: (1) idade ≥ 18 anos e serem moradores da cidade do Rio Grande; (2) apresentarem sintomas de isquemia miocárdica aguda nas últimas 72 horas; (3) apresentarem eletrocardiograma na admissão com supradesnivelamento do segmento ST (SST) com ≥ 1 mm em duas ou mais derivações contíguas periféricas (≥ 2 mm em derivações precordiais) ou novo ou presumivelmente novo bloqueio de ramo esquerdo; e (4) elevação dos marcadores de necrose miocárdica (troponina ou CK-MB).

Os pacientes que não tiveram marcadores de necrose miocárdica mensurados foram incluídos desde que apresentassem sintomas típicos de isquemia miocárdica aguda associados a SST inequívoco que justificassem imediata terapia de reperfusão. Os pacientes que apresentaram novos STEMI durante o período do estudo foram incluídos como um novo evento desde que ocorresse 28 dias após o primeiro evento.

Foram excluídos os pacientes com SST transitório, que, neste estudo, foi definido como resolução espontânea associada à melhora da dor antes do início da terapia de reperfusão. Os reinfartos,^8^ definidos como novos episódios até 28 dias do evento incidente, também foram excluídos, contribuindo apenas para o seguimento clínico.

### Cálculo amostral

Com o objetivo de calcular o tamanho de amostra para a taxa de hospitalizações, foram utilizados os seguintes parâmetros: taxa esperada de 100 casos/100.000 habitantes/ano,^9^ precisão de 20 casos/100.000 habitantes/ano e nível de confiança de 95%. Esse processo resultou em um total de 95.941 indivíduos – a população-alvo deste estudo é de cerca de 160.000 habitantes (Censo Demográfico, 2010). Para o cálculo do tamanho de amostra para mortalidade hospitalar, foram empregados os seguintes parâmetros: proporção esperada de 10%;^9^ precisão de 2,5 pontos percentuais; e nível de confiança de 95%. Esse processo totalizou 554 pacientes. Com base na taxa de hospitalizações esperada de 100/100.000/ano e na população-alvo de 160.000 indivíduos, seriam necessários 4 anos para alcançar a amostra calculada.

### Coleta de dados

**As informações coletadas foram:**

- Sociodemográficas – idade, sexo, atendimento pelo Sistema Único de Saúde (SUS) e classe econômica conforme o Critério de Classificação Econômica do Brasil da Associação Brasileira de Empresas de Pesquisa (ABEP).^10^ Tal classificação, que se baseia no acúmulo de bens materiais em casa e na escolaridade do chefe da família, compreende cinco classes econômicas: A (nível mais alto), B, C, D e E (nível mais baixo).
- História médica – índice de massa corporal (IMC) (calculado por meio de altura e peso autorreferidos); tabagismo (avaliado por meio do relato do paciente ou familiar e definido como ter fumado pelo menos um cigarro no último mês); hipertensão arterial sistêmica, dislipidemia e diabetes (avaliados por meio do relato do paciente ou do familiar mediante diagnóstico médico); e história de infarto prévio, de intervenção coronariana percutânea e de cirurgia de revascularização do miocárdio.
- *Status* clínico na admissão hospitalar – sintoma principal (sintoma que motivou o paciente a procurar a emergência do hospital) e seu intervalo (período entre o início do sintoma até a admissão); frequência cardíaca, pressão arterial sistêmica, classificação de Killip, bloqueio atrioventricular total, topografia da isquemia miocárdica e creatinina sérica.
- Abordagem terapêutica – terapia de reperfusão (fibrinolítico ou intervenção coronariana percutânea), motivos da não reperfusão e medicação adjunta nas primeiras 48 horas.
- Desfechos clínicos hospitalares – morte, reinfarto, choque cardiogênico, arritmia ventricular, complicações mecânicas, acidente vascular cerebral e sangramento conforme os critérios BARC.^11^
- Eventos cardiovasculares dentro do primeiro ano após alta hospitalar – morte cardiovascular, infarto agudo do miocárdio ou acidente vascular cerebral.

Com o propósito de identificar os pacientes elegíveis admitidos no hospital de referência do estudo, uma enfermeira especializada em cardiologia e previamente treinada elaborava diariamente uma lista desses pacientes que chegavam ao setor de emergência. Posteriormente, um cardiologista revisava os potenciais casos e os selecionava conforme os critérios de elegibilidade. As características sociodemográficas e a história médica eram registradas pela enfermeira no momento da internação hospitalar ou assim que possível. O *status* clínico, a abordagem terapêutica e os desfechos clínicos eram avaliados por um cardiologista mediante acompanhamento diário do paciente e revisão do prontuário.

Para a avaliação da ocorrência de eventos cardiovasculares do primeiro ano de seguimento, foi realizado contato telefônico com o paciente 1 ano após a alta hospitalar e, quando apropriado, registros hospitalares foram verificados. Em caso de não respondentes, uma visita em domicílio era realizada. Quando as informações não podiam ser coletadas diretamente com os pacientes, efetuava-se contato com familiar ou pessoa mais próxima.

As informações das hospitalizações eram registradas em formulários impressos e posteriormente digitadas com o uso do programa Microsoft Access.^12^ O controle de qualidade envolveu revisão desses formulários e checagem de amplitude e de consistência dos dados.

### Análise estatística

Para o cálculo da incidência de hospitalizações por STEMI (casos por 100.000 habitantes por ano), o número de hospitalizações constituiu o numerador e a população do Rio Grande (Censo Demográfico 2010), o denominador. Para as análises ajustadas da incidência de hospitalizações, foi empregada a regressão de Poisson.

Os dados das hospitalizações foram sumarizados em frequência e porcentagem para variáveis categóricas e, no caso para variáveis contínuas, em média/desvio padrão ou mediana/percentis de acordo com a normalidade dos dados (verificação da distribuição por meio do teste de Shapiro-Wilk). Para a análise ajustada da mortalidade hospitalar, foi utilizado o modelo linear generalizado (família binominal). Para a análise de sobrevida, utilizou-se o método Kaplan-Meier e, para as análises ajustadas, a regressão de Cox. Todas as análises foram ajustadas para medidas repetidas (um paciente com mais de uma hospitalização)^13^ e conduzidas no programa Stata – versão 14.0.^14^ Um valor de p menor que 0,05 foi considerado para indicar significância estatística.

## Resultados

Durante o período do estudo, ocorreram 575 admissões por sintomas de isquemia miocárdica aguda nas últimas 72 horas associados a SST ao ECG. Dessas, 41 foram excluídas porque os pacientes apresentaram SST transitório enquanto quatro foram descartadas por se tratar de reinfarto. Não houve perdas durante o recrutamento.

### Incidência de hospitalizações

A incidência anual de hospitalização por STEMI no Rio Grande foi de 108 casos por 100.000 habitantes com idade igual ou acima de 25 anos (Tabela 1). A maior taxa foi observada entre homens pertencentes à faixa etária entre 65 e 74 anos. A análise ajustada por sexo mostrou aumento da taxa de hospitalizações conforme aumento da faixa etária (p para tendência linear < 0,001). Em comparação com os pacientes mais jovens, o risco de hospitalização foi 8,9 vezes maior (IC95% 3,5 – 22,7) no grupo etário de 35 a 44 anos, 28,8 (IC95% 11,8 – 70,6) no grupo etário de 45 a 54 anos e 64,9 (IC 95% 26,9 – 156,9) naqueles com 55 anos de idade ou mais.

**Tabela 1:** Taxa anual de hospitalização por infarto agudo do miocárdio com supradesnivelamento do segmento de ST em adultos na cidade de Rio Grande/RS (2011 a 2014)

		Homens			Mulheres			Total
Idade (anos)	População	Número de Casos	Taxa (100.000 hab/ano)	População	Número de Casos	Taxa (100.000 hab/ano)	Número de Casos	Taxa (100.000 hab/ano)
25 – 34	15.609	2	3	16.068	3	5	5	4
35 – 44	12.550	24	48	13.238	12	23	36	35
45 – 54	12.485	84	169	14.087	34	60	118	111
55 – 64	9.486	142	377	10.633	53	125	195	243
65 – 74	4.601	79	433	6.083	27	111	106	249
≥ 75	2.619	33	317	5.158	37	180	70	226
Total	57.350	364	159	65.267	166	64	530	108

As incidências anuais de hospitalizações em homens e mulheres foram 159 e 64 casos por 100.000 habitantes, respectivamente. A incidência ajustada por idade foi 2,8 vezes maior em homens que em mulheres (IC95% 2,3 – 3,3; p < 0,001).

### Dados das hospitalizações

Um total de 522 pacientes experimentaram 530 hospitalizações por STEMI – seis pacientes tiveram duas hospitalizações e um paciente teve três hospitalizações. A procura direta pelo hospital foi de 74% e o atendimento pelo SUS alcançou 85% das internações.

As características sociodemográficas e a história médica estão demonstradas na Tabela 2. A maioria dos pacientes tinha idade igual ou acima de 55 anos, era do sexo masculino e pertencia à classe econômica C. Quase 50% dos pacientes eram tabagistas, 59% tinham hipertensão arterial e 25% apresentavam diabetes melito.

**Tabela 2:** Características sociodemográficas e clínicas dos indivíduos hospitalizados por infarto agudo do miocárdio com supradesnivelamento do segmento de ST (n = 530)

Características sociodemográficas		
Idade (anos), média (desvio padrão)		60,4 (11,6)
Idade (anos), n (%)		
	25 – 44	41 (7,7)
	45 – 54	118 (22,3)
	55 – 64	195 (36,8)
	65 – 74	106 (20,0)
	≥ 75	70 (13,2)
Sexo masculino, n (%)		364 (68,7)
Nível econômico (ABEP), n (%)		
	Classes A e B (mais altos níveis)	179 (33,8)
	Classe C	268 (50,6)
	Classes D e E (mais baixos níveis)	83 (15,6)
**História médica**		
Sobrepeso, n (%) (n = 474)		207 (43,7)
Obesidade, n (%) (n = 474)		112 (23,6)
Tabagismo, n (%)		235 (44,3)
Hipertensão arterial sistêmica, n (%) (n = 500)		295 (59,0)
Diabetes, n (%) (n = 470)		121 (25,7)
Dislipidemia, n (%) (n = 456)		203 (44,5)
Infarto do miocárdio prévio, n (%)		103 (19,9)
Revascularização coronariana prévia (cirúrgica e/ou percutânea), n (%)		67 (12,6)

*ABEP: Associação Brasileira de Empresas de Pesquisa.*

As características da admissão são descritas na Tabela 3. Cerca de 65% dos pacientes chegaram dentro de 3 horas do início dos sintomas, enquanto 94% chegaram dentro de 12 horas. A maioria dos pacientes foi admitida em Killip I e aproximadamente 4% em Killip IV. O infarto inferior (e inferoposterior) foi responsável por metade dos casos enquanto o anterior extenso representou 16%.

**Tabela 3:** Características da admissão hospitalar dos indivíduos hospitalizados por infarto agudo do miocárdio com supradesnivelamento do segmento de ST (n = 530)

Intervalo do sintoma (horas), n (%)		
	0 – 3	342 (64,6)
	> 3 – 6	79 (14,9)
	> 6 – 12	76 (14,3)
	> 12 – 24	17 (3,2)
	> 24 – 72	16 (3,0)
Frequência cardíaca > 100 bpm, n (%) (n = 524)		72 (13,7)
PAS ≥ 180 mmHg e/ou PAD ≥ 110 mmHg, n (%) (n = 516)		114 (26,2)
Classificação de Killip à admissão, n (%) (n = 527)		
	Killip I	463 (87,8)
	Killip II	31 (5,9)
	Killip III	10 (1,9)
	Killip IV	23 (4,4)
Localização do infarto (ECG), n (%)		
	Septal, anteroapical e lateral	140 (26,4)
	Anterior extenso	85 (16,0)
	Inferior e inferoposterior	271 (51,2)
	Posterior	32 (6,0)
	Bloqueio de ramo esquerdo novo	2 (0,4)
Bloqueio AV total, n (%)		21 (4,0)
Creatinina (mg/dL) à admissão, mediana (intervalo interquartil) (n = 516)		0,97 (0,80 – 1,20)

As informações sobre a abordagem terapêutica são apresentadas na Tabela 4. A terapia de reperfusão foi implementada em 80,9% dos pacientes. Quarenta e quatro pacientes (8,3%) não foram considerados elegíveis para terapia de reperfusão, em consequência, na sua maioria, da admissão com mais de 12 horas do início dos sintomas. Entre os pacientes elegíveis, a terapia de reperfusão foi realizada em 88,3%, e a intervenção coronariana percutânea primária (ICP primária) foi o método preferencial. Não foram submetidos à terapia de reperfusão 11,7% dos pacientes elegíveis em virtude, principalmente, de motivos desconhecidos. Quase todos os pacientes receberam dupla antiagregação plaquetária e nenhum deles recebeu inibidor da glicoproteína IIb/IIIa. Com relação à ICP primária, o uso do acesso radial foi de 69,3% e o sucesso angiográfico alcançou 94,7%.

**Tabela 4:** Abordagem terapêutica dos indivíduos hospitalizados por infarto agudo do miocárdio com supradesnivelamento do segmento de ST (n = 530)

Não elegíveis para terapia de reperfusão, n (%)		44 (8,3)
	Intervalo do sintoma > 12 horas, n (%)	42 (95,5)
	Óbito antes da estratégia, n (%)	2 (0,5)
Elegíveis submetidos à terapia de reperfusão, n (%)		429 (88,3)
	ICP primária	356 (83,0)
	ICP primária intencionada, mas não realizada*	28 (6,5)
	Fibrinolítico	45 (10,5)
Elegíveis não submetidos à terapia de reperfusão, n (%)		57 (11,7)
	Motivo desconhecido	50 (88,0)
	Reação alérgica ao trombolítico	6 (10,3)
	Sangramento ativo	1 (1,7)
Acesso transradial na ICP primária, n (%)		266 (69,3)
Sucesso angiográfico na ICP primária, n (%)		337 (94,7)
Medicação nas primeiras 48 horas, n (%)		
	AAS	523 (98,7)
	Clopidogrel	523 (98,7)
	Heparina não fracionada ou heparina de baixo peso	439 (82,8)
	Estatina	487 (91,9)
	Betabloqueador	411 (77,6)
	IECA ou ARA	379 (71,5)

*ICP: intervenção coronariana percutânea; IECA: inibidores da enzima conversora; ARA: antagonistas dos receptores da angiotensina.*

* *Todos pacientes apresentavam fluxo coronariano TIMI 3. Motivos da não realização: opção por cirurgia de revascularização eletiva, opção por ICP eletiva, estenose da lesão-alvo < 50%, vaso-alvo fino e óbito.*

Os desfechos clínicos hospitalares estão demonstrados na Tabela 5. Houve 3% de reinfarto durante a internação hospitalar, quase todos em decorrência de trombose de *stent*. O choque cardiogênico à admissão e na evolução hospitalar totalizou 9%. Ocorreram menos de 1% de complicações mecânicas, de sangramentos e de acidentes vasculares cerebrais isquêmicos. Com relação ao tempo de internação hospitalar, observou-se uma mediana de 7 dias (intervalo interquartil de 6 a 10).

**Tabela 5:** Desfecho clínicos hospitalares em indivíduos hospitalizados por infarto agudo do miocárdio com supradesnivelamento do segmento de ST (n = 530)

Morte, n (%)		47 (8,9)
	Morte cardíaca	38 (7,2)
	Morte não cardíaca (sepse)	9 (1,7)
Reinfarto, n (%)		16 (3,0)
	Trombose de *stent*, n (%)	15 (2,8)
	Trombose pós-uso isolado de extrator de trombo, n (%)	1 (0,2)
Choque cardiogênico (à admissão ou na evolução hospitalar)		48 (9,1)
Fibrilação ventricular ou taquicardia ventricular (na evolução hospitalar)		13 (2,5)
Ruptura de músculo papilar mitral, n (%)		1 (0,2)
Ruptura de septo interventricular, n (%)		1 (0,2)
Ruptura de parede livre, n (%)		1 (0,2)
Sangramento, n (%)		5 (0,9)
	BARC Tipo 2	2 (0,4)
	BARC Tipo 3a	3 (0,6)
Acidente vascular encefálico isquêmico, n (%)		5 (0,9)

A mortalidade hospitalar foi de 8,9%. As taxas de mortalidade conforme grupo etário, sexo e nível socioeconômico, assim como as análises brutas e ajustadas, estão apresentadas na Tabela 6. A mortalidade ajustada por sexo e nível econômico foi maior entre os mais velhos (p para tendência linear = 0,002) alcançando um risco relativo de 3,72 (IC 95%: 1,57 – 8,82) naqueles com 75 anos ou mais. Embora as estimativas ajustadas indiquem maior mortalidade entre as mulheres (risco relativo 1,21; IC95%: 0,69 – 2,14; p = 0,50) e aqueles pertencentes aos mais baixos níveis econômicos (risco relativo 1,66; IC95%: 0,72 – 3,85; p = 0,24), essas diferenças não foram estatisticamente significativas. A mortalidade em 30 dias foi de 9,1%.

**Tabela 6:** Análise bruta e ajustada da mortalidade hospitalar de acordo com idade, sexo e nível econômico

		Mortalidade	Análise bruta		Análise ajustada	
			RR (IC95%)	Valor p	RR (IC95%)	Valor p
Idade, anos						
	25 – 54	5,0%	1,0	< 0,001*	1,0	0,002*
	55 – 64	6,7%	1,33 (0,56 – 3,12)		1,35 (0,58 – 3,16)	
	65 – 74	10,4%	2,06 (0,86 – 4,97)		2,01 (0,83 – 4,90)	
	≥ 75	21,4%	4,26 (1,89 – 9,60)		3,72 (1,57 – 8,82)	
						
Sexo						
	Masculino	7,7%	1,0	0,16†	1,0	0,50†
	Feminino	11,5%	1,49 (0,85 – 2,59)		1,21 (0,69 – 2,14)	
						
Nível econômico						
	Classe A e B	6,2%	1,0	0,03*	1,0	0,24*
	Classe C	9,0%	1,46 (0,73 – 2,90)		1,34 (0,68 – 2,64)	
	Classe D e E	14,5%	2,35 (1,08 – 5,11)		1,66 (0,72 – 3,85)	

RR: risco relativo; IC: intervalo de confiança.

* Teste de Wald para tendência linear.

† Teste de Wald para heterogeneidade.

### Seguimento clínico

Informações de 13 entre os 475 pacientes considerados para o seguimento clínico não foram encontradas, o que representa uma perda de seguimento de 2,7%.

**Figura 1:**
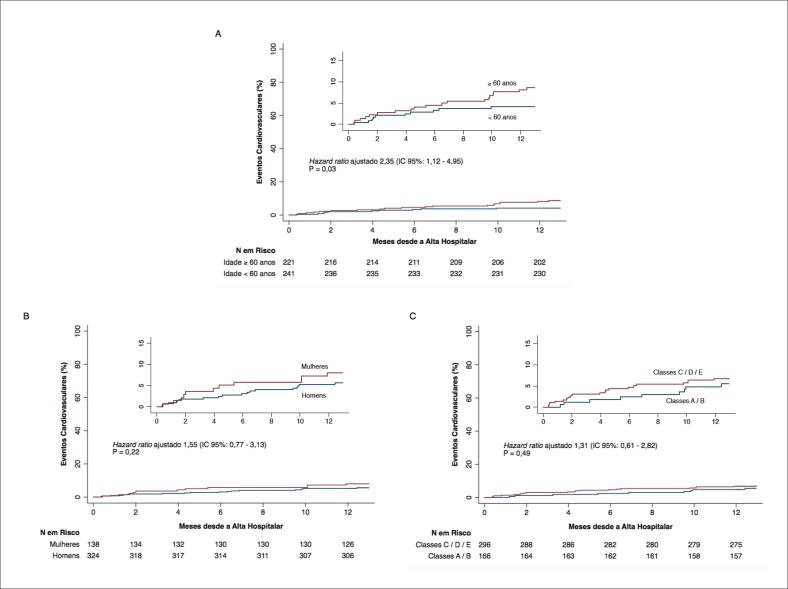
Incidência acumulativa de eventos cardiovasculares (morte cardiovascular, infarto, acidente vascular cerebral) em 1 ano de seguimento após alta hospitalar por STEMI de acordo com idade (Painel A), sexo (Painel B) e nível econômico (Painel C).

A incidência cumulativa de eventos cardiovasculares no final do primeiro ano de seguimento após alta hospitalar por STEMI foi de 6,1% (morte cardiovascular, 3,0%; infarto agudo do miocárdio, 2,4%; e acidente vascular cerebral, 0,7%). A incidência ajustada de eventos cardiovasculares foi maior entre os pacientes com idade igual ou acima de 60 anos (*hazard ratio* 2,35; IC 95%: 1,12 – 4,95; p = 0,03) (Figura 1 – Painel A). A incidência de eventos cardiovasculares ajustada também foi maior entre as mulheres (*hazard ratio* 1,55; IC 95%: 0,77 – 3,13; p = 0,22) e entre aqueles pacientes pertencentes aos mais baixos níveis econômicos (*hazard ratio* 1,31; IC 95%: 0,61 – 2,82; p = 0,49). No entanto, essas diferenças não atingiram significância estatística (Figura 1 – Painéis B e C). Todas essas estimativas foram ajustadas por idade, sexo, nível econômico e também pela presença de cardiopatia isquêmica prévia, que foi definida, neste estudo, como história de infarto do miocárdio e/ou de revascularização miocárdica (cirúrgica e/ou percutânea). A incidência cumulativa de revascularizações não planejadas (cirúrgicas ou percutâneas) no mesmo período de seguimento foi de 4,7%.

## Discussão

A taxa anual de hospitalização por STEMI foi de 108 casos por 100.000 habitantes; essa taxa foi maior entre os homens com mais de 65 anos de idade. A mortalidade hospitalar e a incidência acumulativa de eventos cardiovasculares em 1 ano foram, respectivamente, 8,9% e 6,1%. Ambas as ocorrências foram maiores entre os mais velhos.

A taxa anual de hospitalização por STEMI observada neste estudo foi superior às encontradas em países desenvolvidos. Nos EUA, onde tem havido diminuição dessa incidência ao longo do tempo,^3^ observaram-se taxas iguais a 77 casos por 100.000 habitantes em 2005^4^ e 50/100.000 em 2008.^3^^,^^15^ Na Europa, muitos países também apresentaram taxas de hospitalização por STEMI inferiores à encontrada nesse estudo.^9^ Por outro lado, um estudo conduzido em uma cidade da América Latina obteve uma taxa igual a 90 casos por 100.000 habitantes,^16^ próxima à observada no presente estudo. A maior incidência de hospitalização por STEMI em países em desenvolvimento pode decorrer de pior controle dos fatores de risco^2^ e menor acesso e aderência à medicação.^17^ No que diz respeito à maior taxa de hospitalização observada entre os homens e os indivíduos mais velhos, resultados similares também foram encontrados em outros estudos.^18^^,^^19^

A taxa de terapia de reperfusão foi próxima às encontradas em países desenvolvidos^9^^,^^20^ e superior às observadas em registros nacionais. Esses registros hospitalares que, na maioria, assistem pacientes do sistema público de saúde, mostraram taxas de reperfusão entre 40 e 56%.^21^^–^^23^ Contudo, ainda há uma parcela considerável de pacientes não submetidos à reperfusão, um fato que decorreu principalmente de causas modificáveis. O atraso na busca de assistência médica, bem como o não reconhecimento do quadro clínico de STEMI, são fatores possíveis de melhoria por meio de educação.

No que se refere à mortalidade hospitalar por STEMI, observa-se que varia de acordo com o registro e o país: em registros brasileiros, a mortalidade variou entre 8 e 14%^21^^,^^22^^,^^24^^,^^25^ e, em registros latino-americanos, entre 8 e 11%.^26^^–^^30^ O mesmo cenário pode ser observado na Europa, onde registros conduzidos em vários países evidenciaram taxas oscilando entre 4 e 13%.^9^^,^^31^ Nos EUA, dois registros mostraram taxas iguais a 5,1%^15^ e 9,7%.^4^ Diante desses registros, selecionados de forma não sistemática, a mortalidade por STEMI no presente estudo ficou abaixo dos limites superiores dessas variações. A taxa de reperfusão e o uso da intervenção coronária percutânea por via radial como método preferencial podem ter contribuído para esse resultado.

No entanto, possíveis causas para as variações das taxas de mortalidade entre os estudos devem ser levadas em consideração ao realizar tal análise. As variações das taxas de mortalidade observadas entre os registros podem decorrer do processo metodológico: a) a amostragem de base populacional com registro consecutivo tem menor risco de viés de seleção; b) seleção apenas dos indivíduos que experimentam o primeiro episódio de infarto; c) intervalo do sintoma curto (≤ 12 horas) exclui pacientes com maior risco de morte; d) estudos conduzidos em hospitais com nível terciário de assistência ou em unidades de cuidados intensivos tendem a arrolar pacientes mais graves. Outras causas importantes dessas variações podem ocorrer devido à porcentagem de pacientes submetidos à terapia de reperfusão e ao método dessa terapia utilizado (fibrinolítico ou intervenção coronária percutânea).

A maior mortalidade hospitalar e a maior ocorrência de eventos cardiovasculares em 1 ano de seguimento observada entre os indivíduos mais velhos e do sexo feminino são conhecidas.^32^^,^^33^ A associação entre esses desfechos e os mais velhos também foi identificada neste estudo. Entretanto, a associação com o sexo feminino não foi estatisticamente significativa – uma justificativa para não detecção pode ser a falta de poder do estudo.

A associação entre nível socioeconômico e desfechos cardiovasculares também é conhecida.^34^^,^^35^ Indivíduos com pior condição socioeconômica (baixa escolaridade e baixa renda) tendem a apresentar maior morbimortalidade cardiovascular; essa associação pode ser observada em estudos locais.^36^^–^^39^ Do mesmo modo, o presente estudo mostrou maior mortalidade hospitalar e maior incidência de eventos cardiovasculares em um ano entre aqueles pertencentes aos mais baixos níveis socioeconômicos, porém sem significância estatística. Mais uma vez, a falta de poder pode ter influenciado o resultado.

A principal força deste estudo foi a natureza populacional do registro, uma vez que ela permitiu estimar de modo não enviesado a taxa de hospitalização e a mortalidade por STEMI, bem como a ocorrência de eventos cardiovasculares em 1 ano. O recrutamento consecutivo e sem perdas também contribuiu para diminuir a possibilidade de viés de seleção. Outro aspecto relevante que favoreceu as estimativas diretas foi o recrutamento de pacientes com intervalo de até 72 horas, uma vez que indivíduos que se apresentam com maior tempo de dor têm maior risco de morte. Por fim, destaca-se a pequena taxa de perdas na avaliação do seguimento clínico no final do primeiro ano após a alta hospitalar.

Há de se considerar também que este estudo apresenta algumas limitações. Não houve avaliação do intervalo entre o diagnóstico do STEMI e a terapia de reperfusão; esse dado é importante na avaliação da qualidade do atendimento ao paciente com STEMI. Entretanto, dados desse mesmo hospital no período 2005 a 2007 evidenciaram uma mediana de tempo porta-balão igual a 70 minutos (dados não publicados). Outra limitação foi o seguimento clínico obtido por meio de contato telefônico, fato que impediu uma avaliação objetiva dos eventos. No entanto, como o hospital deste estudo é o centro de referência, procurou-se em registros médicos os eventuais eventos cardiovasculares ocorridos.

Como destacado anteriormente, registros são de fundamental importância. Desse modo, futuros registros devem, com a finalidade de obter estimativas não enviesadas e de comparar com estudos de outros países, ter representatividade da população (seleção randomizada ou inclusão de todos os centros de saúde) e recrutamento consecutivo.^5^ Além disso, recomenda-se selecionar pacientes com um maior tempo de intervalo do sintoma (pelo menos 48 horas).

## Conclusão

Este registro demonstra uma abordagem terapêutica e uma mortalidade hospitalar semelhante às observadas em países desenvolvidos. Entretanto, a taxa de hospitalizações foi maior em comparação a esses países.
