# Supplementary material for: Hospitalização por Infarto Agudo do Miocárdio: Um Registro de Base Populacional
Source: Arq Bras Cardiol. 2020 Nov 1;115(5):916–24. [Article in Portuguese] doi: 10.36660/abc.20190573 (PMC8452198; doi:10.36660/abc.20190573)
Supplement: Translation

# Translation: Hospitalization for Acute Myocardial Infarction: A Population-Based Registry

**Authors:** Leonardo Alves, Carisi Anne Polanczyk

**Affiliations:** 1 Porto AlegreRS Brazil Universidade Federal do Rio Grande do Sul, Porto Alegre, RS – Brazil; 2 Rio GrandeRS Brazil Universidade Federal do Rio Grande, Rio Grande, RS - Brazil; 3 Rio GrandeRS Brazil Hospital Santa Casa do Rio Grande - Hospital de Cardiologia, Rio Grande, RS - Brazil; 4 Porto AlegreRS Brazil Hospital de Clínicas de Porto Alegre, Porto Alegre, RS – Brazil

**Keywords:** Myocardial/mortality, Hospitalization, Epidemiology, Risk Factors, Prevention and Control, Percutaneous Coronary Intervention

## Abstract

**Background:**

ST-segment elevation myocardial infarction (STEMI) is one of the main clinical manifestations of ischemic heart disease. Population-based data are relevant to better understand the current epidemiology of this condition.

**Objective:**

To describe the incidence, therapeutic management, hospital clinical outcomes and cardiovascular events in the first year of follow-up of individuals hospitalized for STEMI.

**Methods:**

Population-based prospective cohort study with consecutive registries of hospitalization for STEMI in a city in southern Brazil from 2011 to 2014. It included patients with STEMI who presented acute myocardial ischemia symptoms in the last 72 hours. A p-value < 0.05 was considered significant.

**Results:**

The annual incidence of STEMI hospitalizations was 108 cases per 100,000 inhabitants. Adjusted incidence was higher among older individuals (relative risk 64.9; 95% CI 26.9–156.9; p for linear trend < 0.001) and among men (relative risk 2.8; 95% CI 2.3–3.3; p < 0.001). There were 530 hospitalizations in the period under evaluation and the reperfusion rate reached 80.9%. Hospital mortality and the one-year follow-up cardiovascular event rate were, respectively, 8.9% and 6.1%. The oldest patients had higher hospital mortality (relative risk 3.72; 95% CI 1.57–8.82; p for linear trend = 0.002) and more one-year follow-up cardiovascular events (hazard ratio 2.35; 95% CI 1.12–4.95; p = 0.03).

**Conclusion:**

This study shows that both the therapeutic approach and hospital mortality are similar to the ones found in developed countries. However, the hospitalization rate was higher in these countries.

## Introduction

Cardiovascular diseases (CVD) are the main cause of mortality in adult males and females and the leading cause of premature death worldwide. Regarding the latter, about 75% occur in low- and middle-income countries.^1^ In Brazil, even though there has been a declining trend, CVD are also the main cause of death in adults.^2^

Ischemic heart disease is responsible for most deaths caused by CVD. The World Health Organization (WHO) estimates that 7.4 million out of 17.7 million people who died of CVD in 2015 had ischemic heart disease. In Brazil, it is also the leading cause of mortality among cardiovascular diseases.^2^

ST-segment elevation myocardial infarction (STEMI) is one of the main clinical manifestations of ischemic heart disease. Its clinical recognition is fundamental so that immediate therapeutic strategies can be drawn up. Studies have shown that, despite decrease in its incidence, mortality related to STEMI has not undergone relevant variations.^3^^,^^4^

In Brazil, there are no population-based data on the hospitalization rate for STEMI. Besides, most information about STEMI hospitalization, such as mortality and reperfusion rate, is collected from registries that have limitations. In general, registries are either restricted to a specific hospital or, when they are multicentric, they do not represent the population, since they result from convenience sampling (invitation or voluntary participation), which may result in biased estimates. Other limitations are non-consecutive recruitment of patients and restrictive eligibility criteria, such as the selection of patients whose symptoms last up to 12 hours (after this period, they are associated with higher mortality).

Therefore, this study aims at describing incidence, therapeutic management, hospital clinical outcomes and cardiovascular events in the first year of follow-up of individuals hospitalized with STEMI in a certain region in southern Brazil. Evaluating these data is relevant not only because this disease has a high incidence in Brazil, but also because there are few population-based studies^5^ in the country. In addition, registries are an efficient way of addressing the implementation of clinical guidelines and databases for healthcare managers, professionals and researchers.^6^^,^^7^

## Methods

### Study design

A prospective cohort study of consecutive registries of hospitalization for STEMI in Rio Grande, RS, Brazil, was carried out from January 2011 to December 2014. The city, located in southern Brazil, has about 200,000 inhabitants, most of whom live in the urban area (Demographic Census, 2010). It has an open emergency service called Hospital de Cardiologia/Santa Casa do Rio Grande, a referral center to treat acute coronary syndromes. Thus, it is the hospital where people whose symptoms suggest that disorder are taken to. As a result, the level of patient referral loss is very low. The city does not have any care line in acute myocardial infarction, which means that patients look for health centers spontaneously.

### Eligibility criteria

To be included in the study, individuals had to meet the following criteria when they were admitted to the hospital: (1) being 18 years old or older and living in Rio Grande, RS; (2) having symptoms of acute myocardial ischemia within 72 hours prior to admission; (3) showing ST-segment elevation (STE) on the electrocardiogram, with ≥ 1 mm in two or more peripheral contiguous leads (≥ 2 mm in precordial leads), or a new, or presumably new, left bundle branch block; and (4) increased markers of myocardial necrosis (troponin or CK-MB).

Patients who did not have their markers of myocardial necrosis measured were included in the study if they had typical symptoms of acute myocardial ischemia associated with unequivocal STE which justified immediate reperfusion therapy. Patients who had new STEMI events throughout the study period were included as new ones, provided that they had occurred at least 28 days after the first one.

Patients with transient STE (defined as spontaneous resolution associated with decreased pain before the beginning of the reperfusion therapy) were excluded from the study. Re-infarction events^8^ (defined as new episodes up to 28 days after the incident one), were also excluded; thus, they only contributed to the clinical follow-up.

### Sample size calculation

The following parameters were used for calculating the sample size of hospitalization rate: expected rate of 100 cases/100,000 inhabitants/year,^9^ precision of 20 cases/100,000 inhabitants/year and 95% confidence level. This process resulted in 95,941 individuals; the target population of this study is about 160,000 inhabitants (Demographic Census, 2010). Parameters used for calculating the sample size of hospital mortality were an expected ratio of 10%,^9^ precision of 2.5 percentage points and 95% confidence level. This process resulted in 554 patients. Based on the expected hospitalization rate of 100/100,000/year and on the target population of 160,000 individuals, four years would be needed to reach the calculated sample.

### Data collection

**The following data were collected:**

- Sociodemographic data — age, sex, medical care at the Brazilian public health system called *Sistema Único de Saúde (SUS)* and economic income class, in line with the Brazilian economic classification criteria, issued by Associação Brasileira de Empresas de Pesquisa (ABEP).^10^ The classification, based on the number of household items and on the householder's education level, comprises five economic classes: A (the highest level), B, C, D and E (the lowest level).
- Medical history — body mass index (based on self-reported height and weight); tobacco smoking (based on the patient's or a family member's report and on the fact that the patient must have smoked at least one cigarette in the month preceding the admission); systemic arterial hypertension, dyslipidemia and diabetes (evaluated by the patient's or a family member's report based on medical diagnosis); and history of prior infarction, percutaneous coronary intervention (PCI) and myocardial revascularization surgery.
- Clinical status at hospital admission — main symptom (the one that made the patient look for the emergency service) and its time interval (period between the beginning of the symptom and admission); heart rate, systemic arterial pressure, Killip classification, complete atrioventricular block, topography of myocardial ischemia and serum creatinine.
- Therapeutic management — reperfusion therapy (fibrinolysis or PCI), reasons for not trying reperfusion and adjunct medication in the first 48 hours.
- Hospital clinical outcomes — death, re-infarction, cardiogenic shock, ventricular arrhythmia, mechanical complications, stroke and bleeding, in line with the criteria issued by the Bleeding Academic Research Consortium (BARC).^11^
- Cardiovascular events in the first year after hospital discharge — cardiovascular death, acute myocardial infarction or stroke.

In order to identify eligible patients admitted to the referral hospital, a registered nurse — specialized in Cardiology and trained to carry out the tasks — kept daily lists of patients who arrived at the emergency service. Afterwards, a cardiologist reviewed potential cases and selected them according to the eligibility criteria. Sociodemographic characteristics and medical history were registered by the nurse when patients were admitted to the hospital. Clinical status, therapeutic management and clinical outcomes were evaluated by a cardiologist who followed patients and reviewed medical records on a daily basis.

To evaluate the occurrence of cardiovascular events in the first year of follow-up, patients were contacted by telephone one year after hospital discharge. When appropriate, medical records were checked. If any of the patients did not answer, household visits were made. When data could not be collected directly from the patients, either their relatives or close connections were contacted.

Hospitalization data were registered on printed forms, then scanned using Microsoft Access.^12^ Quality control comprised form review and checking of data comprehensiveness and consistency.

### Statistical analysis

In order to calculate hospitalization incidence of STEMI (cases per 100,000 inhabitants per year), the number of hospitalization events was the numerator while the city population (Demographic Census, 2010) was the denominator. The Poisson regression model was used for adjusted analyses of hospitalization incidence.

Hospitalization data were summarized into frequency and percentage for categorical variables, and were summarized into mean/standard deviation or median/percentile for continuous variables, depending on data normality (distribution check using the Shapiro-Wilk test). Adjusted analysis of hospital mortality was carried out using the generalized linear model (binomial family). The Kaplan-Meier method was used for the analysis of survival and the Cox regression was applied to adjusted analyses. All analyses were adjusted to repeated measures (a patient with more than one hospitalization event)^13^ and conducted using the Stata program — version 14.0.^14^ To show statistical significance, p was considered below 0.05.

## Results

Throughout the study period, 575 patients were admitted with symptoms of acute myocardial ischemia within 72 hours prior to admission, associated with STE on the electrocardiogram. Forty-five of them were excluded because 41 had transient STE and four underwent re-infarction. There was no loss during recruitment.

### Hospitalization incidence

Annual hospitalization incidence of STEMI in Rio Grande, RS, was 108 cases per 100,000 inhabitants aged 25 or older (Table 1). The highest rate was found among males whose ages ranged from 65 to 74. The analysis adjusted for sex showed that the older the individuals, the higher the hospitalization rate (p for linear trend < 0.001). By comparison with younger patients, hospitalization risk was 8.9-fold higher (95% CI 3.5–22.7) in the group aged 35–44, 28.8-fold higher (95% CI 11.8–70.6) in the group aged 45–54 and 64.9-fold higher (95% CI 26.9–156.9) in the group of individuals who were 55 or older.

**Table 1:** Annual hospitalization rate due to STEMI in adults in Rio Grande, RS, Brazil (2011–2014)

		Males			Females			Total
Age (years)	Population	Number of cases	Rate (100,000 inh./year)	Population	Number of cases	Rate (100,000 inh./year)	Number of cases	Rate (100,000 inh./year)
25 – 34	15,609	2	3	16,068	3	5	5	4
35 – 44	12,550	24	48	13,238	12	23	36	35
45 – 54	12,485	84	169	14,087	34	60	118	111
55 – 64	9,486	142	377	10,633	53	125	195	243
65 – 74	4,601	79	433	6,083	27	111	106	249
≥ 75	2,619	33	317	5,158	37	180	70	226
Total	57,350	364	159	65,267	166	64	530	108

Annual hospitalization incidences in males and females were 159 and 64 cases per 100,000 inhabitants, respectively. Incidence adjusted for age was 2.8-fold higher in males than in females (95% CI 2.3–3.3; p < 0.001).

### Hospitalization data

Data showed that 522 patients underwent 530 hospitalization events due to STEMI — six patients were admitted twice and one patient was admitted three times. Patients going straight to the hospital accounted for 74% of admissions and healthcare conducted at the hospital accounted for 85% of admissions.

Sociodemographic characteristics and medical history are shown in Table 2. Most patients were males who were 55 years old or older and belonged to the economic class C. Almost 50% of patients were smokers, 59% had arterial hypertension and 25% had diabetes mellitus.

**Table 2:** Sociodemographic and clinical characteristics of individuals admitted due to STEMI (n=530)

Sociodemographic characteristics		
Age (years), mean (standard deviation)		60.4 (11.6)
Age (years), n (%)		
	25–44	41 (7.7)
	45–54	118 (22.3)
	55–64	195 (36.8)
	65–74	106 (20.0)
	≥ 75	70 (13.2)
Males, n (%)		364 (68.7)
Economic level (ABEP), n (%)		
	Classes A and B (highest levels)	179 (33.8)
	Class C	268 (50.6)
	Classes D and E (lowest levels)	83 (15.6)
**Medical history**		
Overweight, n (%) (n=474)		207 (43.7)
Obesity, n (%) (n=474)		112 (23.6)
Smoking, n (%)		235 (44.3)
Systemic arterial hypertension, n (%) (n=500)		295 (59.0)
Diabetes, n (%) (n=470)		121 (25.7)
Dyslipidemia, n (%) (n=456)		203 (44.5)
Prior myocardial infarction, n (%)		103 (19.9)
Prior coronary revascularization (surgical and/or percutaneous), n (%)		67 (12.6)

ABEP: Associação Brasileira de Empresas de Pesquisa.

Characteristics of hospital admission are described in Table 3. About 65% of patients arrived within three hours after the onset of symptoms, while 94% of them got there within 12 hours. Most patients were admitted in Killip I and about 4%, in Killip IV. Inferior myocardial infarction (and inferior-posterior myocardial infarction) was responsible for 50% of cases, whereas extensive anterior myocardial infarction represented 16%.

**Table 3:** Characteristics of hospital admission of individuals admitted due to STEMI (n=530)

Interval of symptoms (hours), n (%)		
	0–3	342 (64.6)
	> 3–6	79 (14.9)
	> 6–12	76 (14.3)
	> 12–24	17 (3.2)
	> 24–72	16 (3.0)
Heart rate > 100 bpm, n (%) (n=524)		72 (13.7)
SAP ≥ 180 mmHg and/or DAP ≥ 110 mmHg, n (%) (n=516)		114 (26.2)
Killip classification at admission, n (%) (n=527)		
	Killip I	463 (87.8)
	Killip II	31 (5.9)
	Killip III	10 (1.9)
	Killip IV	23 (4.4)
Location of infarction (ECG), n (%)		
	Septal, anteroapical and lateral	140 (26.4)
	Extensive anterior	85 (16.0)
	Inferior and inferoposterior	271 (51.2)
	Posterior	32 (6.0)
	New left bundle branch block	2 (0.4)
Complete AV block, n (%)		21 (4.0)
Creatinine (mg/dl) at admission, median (interquartile interval) (n=516)		0.97 (0.80–1.20)

Therapeutic management data are shown in Table 4. Reperfusion therapy was performed in 80.9% of patients. Forty-four patients (8.3%) were not considered eligible for reperfusion therapy because admission occurred 12 hours after the onset of symptoms, in most cases. Considering eligible patients, reperfusion therapy was performed in 88.3% and primary percutaneous coronary intervention (PCI) was the preferred method. Reperfusion therapy was not performed in 11.7% of eligible patients; most reasons are unknown. Almost all patients got dual platelet aggregation, but none got any glycoprotein IIb/IIIa inhibitors. Regarding primary PCI, radial access was used in 69.3% and angiographic success was used in 94.7%.

**Table 4:** Therapeutic management of individuals admitted due to STEMI (n=530)

Non-eligible for reperfusion therapy, n (%)		44 (8.3)
	Interval of symptoms > 12 hours, n (%)	42 (95.5)
	Death before the therapy, n (%)	2 (0.5)
Eligible submitted to reperfusion therapy, n (%)		429 (88.3)
	Primary PCI	356 (83.0)
	Planned primary PCI but not performed*	28 (6.5)
	Fibrinolysis	45 (10.5)
Eligible but not submitted to reperfusion therapy, n (%)		57 (11.7)
	Unknown reason	50 (88.0)
	Allergic reaction to fibrinolytics	6 (10.3)
	Active bleeding	1 (1.7)
Radial access at primary PCI, n (%)		266 (69.3)
Angiographic success at primary PCI, n (%)		337 (94.7)
Medication in the first 48 hours, n (%)		
	ASA	523 (98.7)
	Clopidogrel	523 (98.7)
	Unfractionated heparin or low-weight heparin	439 (82.8)
	Statins	487 (91.9)
	Beta blockers	411 (77.6)
	ACEI or ARB	379 (71.5)

PCI: percutaneous coronary intervention; ACEI: angiotensin-converting enzyme inhibitors; ARB: angiotensin receptor blockers.

* All patients were with TIMI grade flow 3. Reasons for not performing: option for elective revascularization surgery, option for elective PCI, target lesion stenosis < 50%, small target vessel and death.

Hospital clinical outcomes are shown in Table 5. There was 3% of re-infarction during hospitalization; almost all cases resulted from stent thrombosis. Cardiogenic shock at admission and while in hospital was 9%. There was less than 1% of mechanical complications, bleedings and ischemic stroke. Concerning length of hospital stay, the median of seven days was found (interquartile range was 6–10 days).

**Table 5:** Hospital clinical outcomes of individuals admitted due to STEMI (n=530)

Death, n (%)		47 (8.9)
	Cardiac death	38 (7.2)
	Non-cardiac death (sepsis)	9 (1.7)
Re-infarction, n (%)		16 (3.0)
	Stent thrombosis, n (%)	15 (2.8)
	Thrombosis after isolated use of thrombus extraction catheter, n (%)	1 (0.2)
	Cardiogenic shock (at admission or during hospitalization)	48 (9.1)
Ventricular fibrillation or ventricular tachycardia (during hospitalization)		13 (2.5)
Mitral papillary muscle rupture, n (%)		1 (0.2)
Ventricular septal rupture, n (%)		1 (0.2)
Left ventricular free wall rupture, n (%)		1 (0.2)
Bleeding, n (%)		5 (0.9)
	BARC Type 2	2 (0.4)
	BARC Type 3a	3 (0.6)
Ischemic stroke, n (%)		5 (0.9)

Hospital mortality was 8.9%. Mortality rates according to age, sex and socioeconomic level, as well as crude and adjusted analyses, are shown in Table 6. Mortality adjusted for sex and economic level was higher among the oldest patients (p for linear trend = 0.002) and achieved a relative risk of 3.72 (95% CI: 1.57–8.82) in those who were 75 years old or older. Even though adjusted estimates showed higher mortality among females (relative risk 1.21; 95% CI: 0.69–2.14; p = 0.50) and individuals who belong to the lowest economic levels (relative risk 1.66; 95% CI: 0.72–3.85; p = 0.24), these differences were not statistically significant. Thirty-day mortality was 9.1%.

**Table 6:** Crude and adjusted analyses of hospital mortality according to age, sex and economic class

		Mortality	Crude analysis		Adjusted analysis	
			RR (95% CI)	P value	RR (95% CI)	P value
Age, years						
	25–54	5.0%	1.0	< 0.001*	1.0	0.002*
	55–64	6.7%	1.33 (0.56–3.12)		1.35 (0.58–3.16)	
	65–74	10.4%	2.06 (0.86–4.97)		2.01 (0.83–4.90)	
	> 75	21.4%	4.26 (1.89–9.60)		3.72 (1.57–8.82)	
						
Sex						
	Male	7.7%	1.0	0.16†	1.0	0.50†
	Female	11.5%	1.49 (0.85–2.59)		1.21 (0.69–2.14)	
						
Economic level						
	Classes A and B	6.2%	1.0	0.03*	1.0	0.24*
	Class C	9.0%	1.46 (0.73–2.90)		1.34 (0.68–2.64)	
	Classes D and E	14.5%	2.35 (1.08–5.11)		1.66 (0.72–3.85)	

RR: relative risk; CI: confidence interval.

* Wald test for linear trend.

† Wald test for heterogeneity.

### Clinical follow-up

Data on 13 out of 475 patients who were considered for clinical follow-up were not found, so follow-up loss was 2.7%.

Cumulative incidence of cardiovascular events at the end of the first year of follow-up, after hospital discharge due to STEMI, was 6.1% (cardiovascular death was 3.0%; acute myocardial infarction was 2.4% and stroke was 0.7%). Adjusted incidence of cardiovascular events was higher among patients who were 60 years old or older (hazard ratio 2.35; 95% CI: 1.12–4.95; p = 0.03) (Figure 1 — Panel A). It was also higher among females (hazard ratio 1.55; 95% CI: 0.77–3.13; p = 0.22) and among individuals that belonged to the lowest economical levels (hazard ratio 1.31; 95% CI: 0.61–2.82; p = 0.49). However, these differences did not have any statistical significance (Figure 1 — Panels B and C). All estimates were adjusted for age, sex, economic level and prior ischemic cardiomyopathy, which was defined as the history of myocardial infarction and/or myocardial revascularization (surgical and/or percutaneous). Cumulative incidence of non-planned revascularization (surgical or percutaneous) in the follow-up period was 4.7%.

**Figure 1:** Cumulative incidence of cardiovascular outcomes (cardiovascular death, infarction, stroke) at the end of the first year of follow-up after hospital discharge due to STEMI based on age (Panel A), sex (Panel B) and economic class (Panel C).

## Discussion

The annual hospitalization rate for STEMI, which was 108 cases per 100,000 inhabitants, was higher among males older than 65. Hospital mortality and one-year cumulative incidence of cardiovascular events were 8.9% and 6.1%, respectively. Both occurrences were higher among the oldest individuals.

The annual hospitalization rate for STEMI found by this study was higher than the ones found in developed countries. In the United States, where there has been a decrease in the incidence over time:^3^ rates of 77 cases per 100,000 inhabitants and 50/100,000 were found in 2005^4^ and in 2008,^3^^,^^15^ respectively. In Europe, many countries also had lower hospitalization rates for STEMI than the one found in this study.^9^ However, a study carried out in a city in Latin America found a rate of 90 cases per 100,000 inhabitants,^16^ which was close to the one of this study. The highest incidence of hospitalization for STEMI in developing countries may result from the facts that they have poor control of risk factors^2^ and their populations have less access and adherence to medication.^17^ Concerning the highest hospitalization rate found among males and older individuals, similar results were also reported by other studies.^18^^,^^19^

The reperfusion therapy rate was close to the one observed in developed countries^9^^,^^20^ and higher than the ones found in national registries. Registries found in hospitals that assist mostly SUS patients showed reperfusion rates ranging from 40% to 56%.^21^^,^^23^ However, there is a considerable number of patients who were not submitted to reperfusion, a fact that resulted mainly from modifiable causes. Delay in seeking medical care and poor recognition of STEMI patients' clinical status are factors that may be improved with higher education levels.

Regarding hospital mortality resulting from STEMI, it depends on the registry and country, i.e., in Brazilian registries, it ranged from 8% to 14%,^21^^,^^22^^,^^24^^,^^25^ while in Latin American registries, it ranged from 8% to 11%.^26^^–^^30^ The same scenario may be observed in Europe, where registries carried out by several countries showed rates that ranged from 4% to 13%.^9^^,^^31^ In the United States, two registries showed rates of 5.1%^15^ and 9.7%.^4^ By comparison with these registries, which were selected in a non-systematic way, mortality due to STEMI, in this study, was below the highest limits of these variations. Reperfusion rate and the use of radial access PCI as the preferred method may have contributed to this result.

However, some causes of variations in mortality rates found by these studies should be considered in this analysis. Variation in mortality rates provided by the registries may result from the methodological process: a) population-based sampling with consecutive registry has low risk of selection bias; b) only individuals that underwent the first infarction are selected; c) time interval of the short symptom (≤ 12 hours) excludes patients at high risk of death; and d) studies carried out either in hospitals that provide tertiary care or in intensive care units tend to register the most severe patients. Other important causes of variations may occur because of the percentage of patients submitted to reperfusion therapy and to its method (fibrinolysis or PCI).

The highest hospital mortality rate and the highest occurrence of cardiovascular events in the 1-year follow-up found among the oldest individuals and females were known.^32^^,^^33^ An association between these outcomes and the oldest individuals was also identified in this study. However, an association with females was not statistically significant; it may not have been detected because the study did not have power.

An association between socioeconomic levels and cardiovascular outcomes was also known.^34^^,^^35^ Individuals with low socioeconomic status (low level of education and low income) tend to be affected by cardiovascular morbimortality, an association that is found in local studies.^36^^–^^39^ Likewise, this study showed that there was high one-year hospital mortality and high one-year incidence of cardiovascular events among individuals who belonged to the lowest socioeconomic levels, but there was no statistical significance. In this case, the fact that the study did not have power may also have influenced its results.

The main strength of this study was its population-based registry, since it enabled an unbiased hospitalization rate and mortality due to STEMI to be estimated, as well as the occurrence of cardiovascular events in one year. Consecutive recruitment, with no loss, also contributed to decrease selection bias. Another relevant issue that favored direct estimates was the recruitment of patients whose time interval was 72 hours, since the ones whose period of pain was longer had higher risk of death. Finally, the low rate of loss in the evaluation of clinical follow-up at the end of the first year after hospital discharge should be highlighted.

Limitations of the study should be considered. The time interval between STEMI diagnosis and reperfusion therapy was not evaluated; this data is important to evaluate the quality of care provided to STEMI patients. However, data collected in the hospital from 2005 to 2007 showed median door-to-balloon time of 70 minutes (unpublished data). Another limitation was the clinical follow-up by phone, which prevented an objective evaluation of events. Since the hospital is a referral center, cardiovascular events that occurred there were investigated in medical records.

As highlighted before, registries are fundamental. Thus, in order to provide unbiased estimates and enable comparison with studies carried out in other countries, future registries should be representative of the population (either randomized selection or inclusion of all health centers) and consecutive recruitment.^5^ Besides, this study recommends that selection should include patients with a longer time interval from the symptom (at least 48 hours).

## Conclusion

This study shows that therapeutic management and hospital mortality in developing countries was similar to both found in developed countries. However, the hospitalization rate was higher in the former.
